# Incidence of type 2 diabetes mellitus in men receiving steroid 5α-reductase inhibitors: population based cohort study

**DOI:** 10.1136/bmj.l1204

**Published:** 2019-04-10

**Authors:** Li Wei, Edward Chia-Cheng Lai, Yea-Huei Kao-Yang, Brian R Walker, Thomas M MacDonald, Ruth Andrew

**Affiliations:** 1 Research Department of Practice and Policy, School of Pharmacy, University College London, London, UK; 2 School of Pharmacy, Institute of Clinical Pharmacy and Pharmaceutical Sciences, College of Medicine, National Cheng Kung University, Tainan City, Taiwan; 3 University/BHF Centre for Cardiovascular Science, Queen’s Medical Research Institute, University of Edinburgh, Edinburgh EH16 4TJ, UK; 4 Institute of Genetic Medicine, International Centre for Life, Newcastle University, Newcastle upon Tyne, UK; 5 Medicines Monitoring Unit, Ninewells Hospital and Medical School, Dundee, UK

## Abstract

**Objective:**

To investigate the incidence of new onset type 2 diabetes mellitus in men receiving steroid 5α-reductase inhibitors (dutasteride or finasteride) for long term treatment of benign prostatic hyperplasia.

**Design:**

Population based cohort study.

**Setting:**

UK Clinical Practice Research Datalink (CPRD; 2003-14) and Taiwanese National Health Insurance Research Database (NHIRD; 2002-12).

**Participants:**

Men in the CPRD who received dutasteride (n=8231), finasteride (n=30 774), or tamsulosin (n=16 270) were evaluated. Propensity score matching (2:1; dutasteride to finasteride or tamsulosin) produced cohorts of 2090, 3445, and 4018, respectively. In the NHIRD, initial numbers were 1251 (dutasteride), 4194 (finasteride), and 86 263 (tamsulosin), reducing to 1251, 2445, and 2502, respectively, after propensity score matching.

**Main outcomes measure:**

Incident type 2 diabetes using a Cox proportional hazard model.

**Results:**

In the CPRD, 2081 new onset type 2 diabetes events (368 dutasteride, 1207 finasteride, and 506 tamsulosin) were recorded during a mean follow-up time of 5.2 years (SD 3.1 years). The event rate per 10 000 person years was 76.2 (95% confidence interval 68.4 to 84.0) for dutasteride, 76.6 (72.3 to 80.9) for finasteride, and 60.3 (55.1 to 65.5) for tamsulosin. There was a modest increased risk of type 2 diabetes for dutasteride (adjusted hazard ratio 1.32, 95% confidence interval 1.08 to 1.61) and finasteride (1.26, 1.10 to 1.45) compared with tamsulosin. Results for the NHIRD were consistent with the findings for the CPRD (adjusted hazard ratio 1.34, 95% confidence interval 1.17 to 1.54 for dutasteride, and 1.49, 1.38 to 1.61 for finasteride compared with tamsulosin). Propensity score matched analyses showed similar results.

**Conclusions:**

The risk of developing new onset type 2 diabetes appears to be higher in men with benign prostatic hyperplasia exposed to 5α-reductase inhibitors than in men receiving tamsulosin, but did not differ between men receiving dutasteride and those receiving finasteride. Additional monitoring might be required for men starting these drugs, particularly in those with other risk factors for type 2 diabetes.

## Introduction

Previous studies suggest that commonly used drugs, such as antihypertensives,^1^ statins,^2^ ^3^ antipsychotics,^4^ antiretrovirals,^5^ immunosuppressants,^6^ and corticosteroids,^7^ increase the risk of type 2 diabetes mellitus. Recent findings^8^ ^9^ show that steroid 5α-reductase inhibitors might also be implicated. 5α-reductase inhibitors are prescribed to treat benign prostatic hyperplasia (BPH), a disease affecting approximately 50% of older men. These drugs are usually prescribed if α blockers have been ineffective or the prostate gland is substantially enlarged.^10^ 5α-reductase inhibitors prevent conversion of testosterone to the more active 5α-dihydrotestosterone and reduce androgen dependent prostate growth.^11^ Two 5α-reductase inhibitors are marketed: finasteride, first in class, which selectively inhibits 5α-reductase 2; and dutasteride, which inhibits 5α-reductase 2 and 5α-reductase 1. Although 5α-reductase 2 is highly expressed in prostate and skin, 5α-reductase 1 is also active in metabolic tissues (liver, adipose, and skeletal muscle).^8^

A recent short term experimental medicine study showed that dutasteride induces insulin resistance, a major risk factor for type 2 diabetes; however, neither finasteride nor the α blocker tamsulosin had this effect.^8^ Moreover, dutasteride administered for three weeks promotes hepatic steatosis, although this result was not found for finasteride.^9^ Traish and colleagues reported increased blood glucose and glycated haemoglobin A1c after approximately three years of dutasteride treatment, but did not assess the effect of finasteride.^12^ These findings are consistent with increased susceptibility to diet induced obesity, impaired glucose tolerance, and fatty liver reported in *Srd5a1* ^-/-^ mice; similar changes were found in obese rats receiving dual 5α-reductase inhibitors.^13^ ^14^ Men requiring drug treatment for BPH are older and more susceptible to type 2 diabetes, so treatments that exacerbate this risk should be avoided. A previous study that reported a lower risk of type 2 diabetes in men receiving 5α-reductase inhibitors did not account for baseline metabolic differences.^15^

We investigated whether treatment with dutasteride or finasteride increases the risk of type 2 diabetes compared with tamsulosin alone in two population based cohorts. We also compared the risk of developing type 2 diabetes after dutasteride treatment and finasteride treatment.

## Methods

### Discovery cohort

We performed a population based cohort study using data from the UK Clinical Practice Research Datalink (CPRD). This database contains anonymised longitudinal medical records from more than 500 primary care practices with 4.4 million active patients. Data have been collected since 1987 and cover approximately 7% of the UK population; these data can be generalised to the whole UK population.^16^

Our study population included men aged at least 40 with a recorded diagnosis of BPH in their general practice notes, or prescribed dutasteride, finasteride, or tamsulosin. These patients were registered in primary care between 2003 and 2014 (Read codes, supplementary table S1A). Patients were followed up until the end of December 2014 and were censored if they experienced an outcome event, died, left their general practice during the study period, or switched drugs. We excluded patients if they had received prescriptions for finasteride or tamsulosin before 2003 or had a history of cancer, diabetes, or oral glucose lowering or insulin treatment before the index date (date of first prescription of dutasteride, finasteride, or tamsulosin).

Our study period was 11 calendar years (2003-14) and we included three cohorts that were identified and followed up in a similar way. The first cohort comprised patients who received at least two prescriptions of dutasteride. These patients entered the study on the date of the first prescription for dutasteride (index date) and were included if they remained in the database for at least 90 days. Patients switching from dutasteride to finasteride or α blockers were censored at that time. We created a separate group of patients concurrently prescribed dutasteride and tamsulosin, but these patients were also included in a “total” cohort along with those prescribed dutasteride alone. A second cohort comprised patients who received at least two prescriptions of finasteride either alone or as a “total” cohort, which included those taking finasteride in combination with tamsulosin. These patients entered the study on the date of the first prescription for finasteride (index date) and were included if they remained in the database for at least 90 days. A third cohort comprised patients who received at least two prescriptions of tamsulosin alone. Tamsulosin was selected as the representative α blocker because it is the most common α blocker prescribed for BPH, which is its sole indication. These patients entered the study on the date of the first prescription for tamsulosin (index date) and were included if they remained in the database for at least 90 days. We excluded patients prescribed tamsulosin without a recorded diagnosis code for BPH (international classification of diseases, ninth revision (ICD-9) code 600).

### Primary outcome and covariates

The primary outcome was the incidence of new onset type 2 diabetes during the follow-up period (Read codes, supplementary table S1B), or prescription of oral glucose lowering drugs or insulin. We compared the incidence of new onset type 2 diabetes in the dutasteride and finasteride cohorts versus the tamsulosin cohort, and the incidence in the dutasteride cohort versus the finasteride cohort. Covariates included age, smoking status, alcohol consumption, body mass index and physical activity at index date, and duration of BPH; history of hypertension, dyslipidaemia, and chronic obstructive pulmonary disease; use of β blockers, diuretics, angiotensin converting enzyme inhibitors, angiotensin receptor blockers, statins, and oral corticosteroids in the previous three years; and health status assessed by the number of outpatient visits three months before and three months after the index date. We defined the duration of BPH from the date of the first record of BPH to the index date. For patients who started drug treatment without a previously recorded diagnosis of BPH, the duration was zero.

### Analysis after propensity matching

We calculated a propensity score for each patient to minimise confounding by indication, when patients with other risk factors for type 2 diabetes might be more likely to receive dutasteride. We used logistic regression to obtain the propensity score, and the covariables were the confounding variables at baseline (age, smoking status, alcohol consumption, body mass index, physical activity, and duration of BPH from first diagnosis; history of chronic obstructive pulmonary disease, hypertension, and dyslipidaemia; health status assessed by number of outpatient visits three months before and three months after the index date; use of diuretics, angiotensin converting enzyme inhibitors, angiotensin receptor blockers, β blockers, statins, and oral corticosteroids in previous three years). Comparator cohorts matched by propensity score (within ±0.05, comprising up to two matched controls for each dutasteride exposed patient) were created using patients prescribed finasteride or tamsulosin from the same practice.

### Replication cohort

The study was replicated using the Taiwanese National Health Insurance Research Database (NHIRD), which is a validated^17^ database with more than 99% of the population registered. The NHIRD includes demographic data, information for healthcare professionals and medical facilities, health service records and expenditure claims from inpatient and ambulatory care, and dispensing data. Further datasets include patients with cancer, diabetes, dental problems, catastrophic illness, and psychiatric disease.

The study period was from 2002 to 2012. The computer software randomly drew three million patients from the total Taiwanese population of 23 million. We used the same criteria for cohorts, participants, and outcome as described for the CPRD database. Only a urologist prescribed finasteride and dutasteride, therefore records on their use were considered specific for BPH. We excluded patients prescribed tamsulosin without a recorded diagnosis code for BPH (ICD-9 code 600).

### Outcome and covariates

We defined the outcome of new onset type 2 diabetes by the first record of type 2 diabetes (ICD-9 codes 250.x0 or 250.x2) or use of at least one oral drug for diabetes. We censored patients in the same way as for the CPRD. Covariates included age and duration of BPH from first diagnosis; history of chronic obstructive pulmonary disease, hypertension, or dyslipidaemia; health status assessed by number of outpatient visits three months before and three months after the index date; and previous one year use (standard assessment of previous drug use in Taiwanese database^18^) of diuretics, statins, angiotensin converting enzyme inhibitors, angiotensin receptor blockers, and oral corticosteroids. We performed propensity matching in the same way as for the CPRD database, but without data on body mass index, smoking status, alcohol consumption, and physical activity.

### Statistical analysis

We summarised data as mean (standard deviation) or median (interquartile range) for continuous variables, and number (percentage) for categorical variables. Data distributions and Cox model assumptions were checked before analysis by using proportionality tests and log-log plots. We carried out χ^2^ tests, one way analysis of variance, and Kruskal-Wallis tests for the baseline characteristics. Person year time for each patient was calculated as the time from the index date to the end of follow-up. We used Cox proportional hazard models before and after propensity matching. In the UK cohort, missing data for body mass index, smoking status, alcohol consumption, and physical activity were categorised into a further group and adjusted in the final model to include all patients. We performed cumulative incidence plots and log rank tests to compare outcomes among the cohorts. Analyses were carried out using SAS software, version 9.4 (Cary, NC).

### Patient and public involvement

This research was performed without patient involvement. We thank the patients for allowing their records to be retained in the databases. We have included patients in our dissemination strategy.

## Results

### Discovery cohort: CPRD

**Fig 1:**
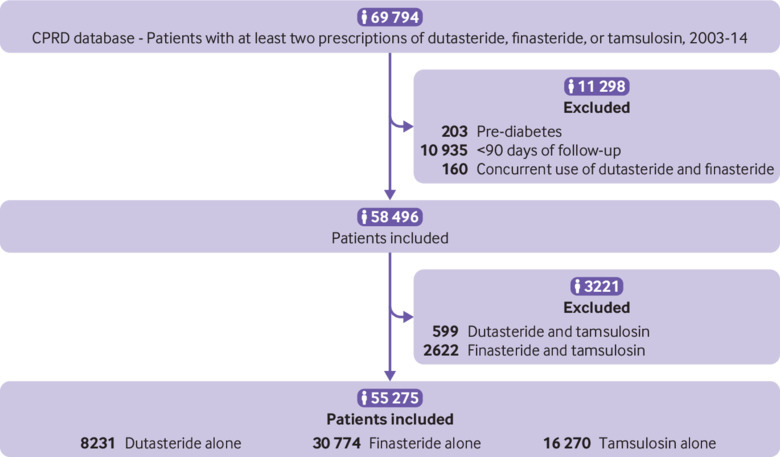
Flow chart showing UK Clinical Practice Research Datalink (CPRD) study cohorts

A total of 69 794 patients received at least two prescriptions of dutasteride, finasteride, or tamsulosin (fig 1). After we applied exclusions, 55 275 participants remained; 39 005 patients using 5α-reductase inhibitors were included (8231 dutasteride and 30 774 finasteride) and 16 270 receiving tamsulosin. At baseline, patients receiving dutasteride or finasteride were older, had more comorbidities, except for dyslipidaemia, and used more oral corticosteroids and cardiovascular drugs than those receiving tamsulosin (table 1). There were fewer differences in baseline characteristics between the dutasteride and finasteride groups, with no differences in body mass index. When we included patients receiving dutasteride combined with tamsulosin or finasteride combined with tamsulosin the numbers increased by 599 and 2622, respectively, but baseline characteristics did not change substantively (supplementary table S2).

**Table 1:** Baseline characteristics for UK Clinical Practice Research Datalink cohorts receiving single drugs before and after propensity matching. Values are means (standard deviations) unless stated otherwise

Characteristics	Before propensity score matching					After propensity score matching			
	Dutasteride (n=8231)	Finasteride (n=30 774)	Tamsulosin (n=16 270)	P value		Dutasteride (n=2090)	Finasteride (n=3445)	Tamsulosin (n=4018)	P value
Age (years)	71.9 (9.8)	72.2 (10.9)	69.1 (10.3)	<0.01	71.3 (9.2)	72.1 (9.1)	71.2 (9.3)	<0.01
Body mass index category*:								
<18.5	46 (0.7)	183 (0.7)	91 (0.6)	0.01	13 (0.7)	17 (0.6)	29 (0.8)	0.48
18.5-25	2443 (34.9)	9028 (34.9)	4794 (33.6)		644 (35.3)	1022 (34.6)	1231 (35.0)	
25-30	3282 (46.9)	11 913 (46.1)	6846 (48.1)		876 (48.1)	1423 (48.1)	1680 (47.7)	
>30	1229 (17.5)	4944 (18.3)	2518 (17.7)		290 (15.9)	496 (16.8)	581 (16.5)	
Smoker*:								
Yes	849 (10.7)	3292 (11.3)	1859 (11.8)	<0.01	221 (10.9)	337 (10.3)	461 (11.9)	0.08
No	3772 (47.6)	13 356 (45.9)	7485 (47.4)		976 (48.3)	1515 (46.1)	1810 (46.5)	
Former	3311 (41.7)	12 447 (42.8)	6458 (40.9)		826 (40.8)	1431 (43.6)	1618 (41.6)	
Physical activity*:								
Inactive	378 (9.8)	1335 (9.8)	699 (9.6)	<0.01	87 (8.6)	122 (7.7)	191 (9.6)	0.04
Gentle	1418 (36.8)	5321 (39.0)	2636 (34.1)		358 (35.4)	628 (39.5)	709 (35.6)	
Moderate	1861 (48.2)	6333 (46.4)	3917 (50.7)		508 (50.2)	764 (48.0)	965 (48.4)	
Vigorous	201 (5.2)	650 (4.8)	473 (6.1)		59 (5.8)	77 (4.8)	129 (65)	
Alcohol consumption*:				<0.01				
Yes	5915 (82.2)	21 879 (82.5)	12 233 (84.4)		1563 (84.8)	2520 (84.0)	3061 (84.8)	0.94
No	1061 (14.7)	3831 (14.4)	1850 (12.8)		232 (12.6)	395 (13.2)	454 (1267)	
Former	220 (3.1)	820 (3.1)	416 (2.9)		49 (2.7)	84 (2.8)	96 (2.7)	
Median (interquartile range) duration of BPH (days)*	14 (0-1476)	13 (0-1384)	56 (27-892)	<0.01	16 (0-1521)	17 (0-1523)	56 (26-1110)	<0.01
Disease history (No (%)):								
COPD	633 (7.7)	2571 (8.4)	1169 (7.2)	<0.01	159 (7.6)	271 (7.9)	332 (8.3)	0.63
Dyslipidaemia	1235 (15.0)	4294 (14.0)	2711 (16.7)	<0.01	342 (16.4)	590 (17.1)	657 (16.4)	0.62
Hypertension	2820 (34.3)	1114 (36.1)	5241 (32.2)	<0.01	717 (34.3)	1217 (35.3)	1457 (36.3)	0.30
Other drug use (No (%))†:								
β blocker	2179 (26.5)	7662 (24.9)	3471 (21.3)	<0.01	538 (25.7)	834 (24.2)	936 (23.3)	0.11
Statin	3277 (39.8)	11 704 (38.0)	5871 (36.1)	<0.01	768 (36.7)	1267 (36.8)	1551 (38.6)	0.18
ACE inhibitor	2246 (27.3)	8644 (28.1)	3844 (23.6)	<0.01	524 (25.1)	913 (26.5)	1063 (26.5)	0.43
ARB	829 (10.1)	2848 (9.3)	1359 (8.4)	<0.01	183 (8.8)	321 (9.3)	397 (9.9)	0.35
Diuretic	2445 (29.7)	9315 (30.3)	3669 (22.6)	<0.01	584 (27.9)	956 (27.8)	1044 (26.0)	0.13
Oral corticosteroid	886 (10.7)	3095(10.1)	1497 (9.2)	<0.01	222 (10.6)	319 (9.3)	420 (10.5)	0.15
No of GP contacts‡	10.5 (2.2)	10.2 (2.3)	10.0 (2.4)	<0.01	10.4 (2.3)	10.1 (2.4)	10.2 (2.3)	<0.01

ACE=angiotensin converting enzyme; ARB=angiotensin receptor blocker; BMI=body mass index; BPH=benign prostatic hyperplasia; COPD=chronic obstructive pulmonary disease; GP=general practitioner.

* Excluding missing data.

† In previous three years.

‡ Three months before or three months after index date.

#### Analysis of main CPRD cohort

We recorded 2081 new onset type 2 diabetes events (368, 1207, and 506 for the dutasteride, finasteride, and tamsulosin groups, respectively) during a mean follow-up of 5.2 (SD 3.1) years (5.9 (3.1), 5.1 (3.2), and 5.2 (3.1) for dutasteride, finasteride, and tamsulosin, respectively). Cohorts receiving dutasteride combined with tamsulosin or finasteride combined with tamsulosin added a further 27 and 82 events, respectively. Therefore, there were 395 and 1289 events in the “total” cohorts for dutasteride and finasteride, respectively.

**Fig 2:**
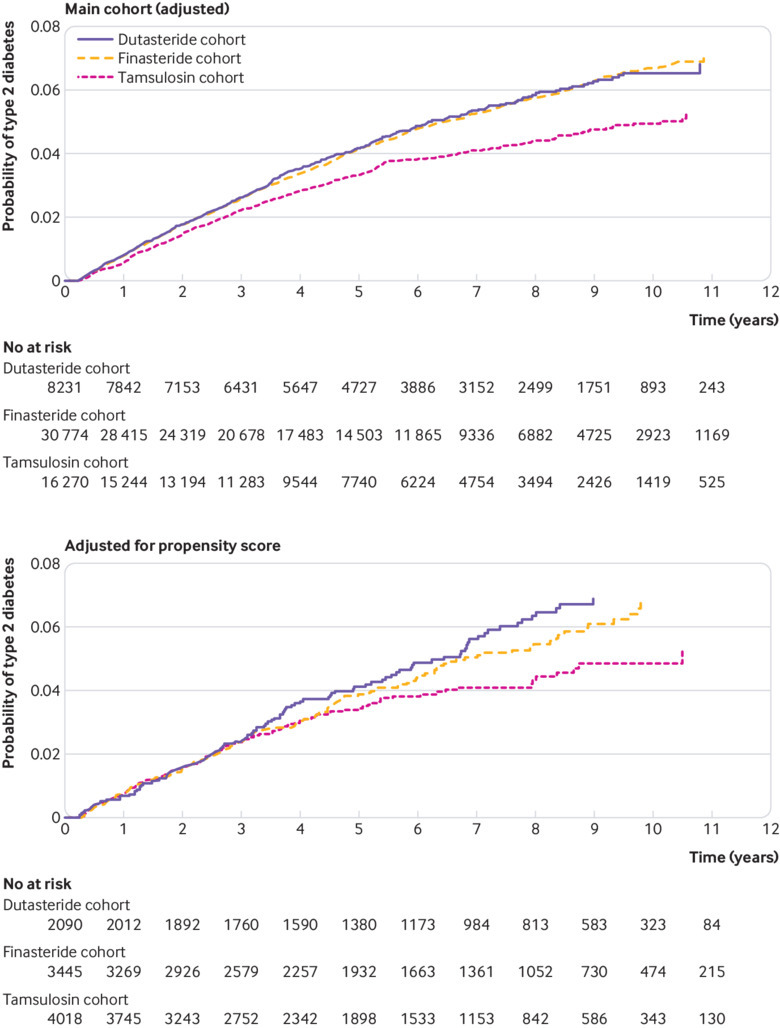
Cumulative incidence of type 2 diabetes in Clinical Practice Research Datalink cohort over study period: main cohort (adjusted), and adjusted for propensity score

The event rate was 76.2 per 10 000 person years (95% confidence interval 68.4 to 84.0) for dutasteride and 76.6 (72.3 to 80.9) for finasteride compared with 60.3 (55.1 to 65.5) for tamsulosin. The cumulative incidence of developing type 2 diabetes (P<0.01) was higher in the dutasteride and finasteride cohorts compared with tamsulosin cohort (fig 2). Similar event rates were observed in the “total” cohorts: 76.6 (69.0 to 84.1) for total dutasteride and 76.1 (71.9 to 80.2) for total finasteride. The risk of developing type 2 diabetes was again higher for patients receiving dutasteride or finasteride compared with tamsulosin (table 2). The increase in risk of type 2 diabetes did not differ among patients receiving dutasteride or finasteride alone or when we included patients receiving these drugs in combination with tamsulosin (table 2; fig 2).

**Table 2:** Hazard ratios of incident type 2 diabetes mellitus in the UK CPRD and Taiwanese NHIRD cohorts

Cohort	Primary analysis			Matched by propensity score
	Unadjusted hazard ratio (95% CI)	Adjusted hazard ratio* (95% CI)		Adjusted hazard ratio (95% CI)
CPRD:				
Finasteride alone *v* tamsulosin alone	1.28 (1.15 to 1.42)	1.26 (1.10 to 1.45)		1.22 (0.95 to 1.57)
Dutasteride alone *v* tamsulosin alone	1.29 (1.13 to 1.48)	1.32 (1.08 to 1.61)		1.34 (1.02 to 1.75)
Dutasteride alone *v* finasteride alone	1.00 (0.89 to 1.13)	1.07 (0.87 to 1.31)		1.08 (0.83 to 1.40)
Total finasteride† *v* tamsulosin alone	1.27 (1.14 to 1.40)	1.22 (1.07 to 1.39)		1.32 (1.06 to 1.64)
Total dutasteride† *v* tamsulosin alone	1.29 (1.13 to 1.48)	1.32 (1.09 to 1.58)		1.34 (1.05 to 1.71)
Total dutasteride† *v* total finasteride†	1.02 (0.91 to 1.14)	1.08 (0.90 to 1.31)		1.04 (0.82 to 1.31)
NHIRD:				
Finasteride alone *v* tamsulosin alone	1.47 (1.36 to 1.59)	1.49 (1.38 to 1.61)		1.61 (1.46 to 1.80)
Dutasteride alone *v* tamsulosin alone	1.55 (1.35 to 1.78)	1.34 (1.17 to 1.54)		1.18 (1.00 to 1.40)
Dutasteride alone *v* finasteride alone	1.06 (0.90 to 1.24)	0.90 (0.77 to 1.06)		0.94 (0.80 to 1.11)
Total finasteride† *v* tamsulosin alone	1.49 (1.39 to 1.60)	1.50 (1.39 to 1.62)		1.48 (1.34 to 1.63)
Total dutasteride† *v* tamsulosin alone	1.32 (1.15 to 1.51)	1.34 (1.17 to 1.53)		1.18 (1.01 to 1.40)
Total dutasteride† *v* total finasteride†	1.05 (0.90 to 1.21)	0.89 (0.77 to 1.03)		0.82 (0.67 to 0.99)

CPRD=Clinical Practice Research Datalink; NHIRD=National Health Insurance Research Database.

* For CPRD, adjusted for age, duration of condition, body mass index, smoking status, alcohol consumption, physical activity, previous medical conditions (chronic obstructive pulmonary disease, dyslipidaemia, and hypertension), number of general practitioner contacts, and use of corticosteroids and cardiovascular drugs; for NHIRD, adjusted for age, duration of condition, previous medical conditions (chronic obstructive pulmonary disease, dyslipidaemia, and hypertension), number of general practitioner contacts, and use of corticosteroids and cardiovascular drugs.

† Cohorts of patients receiving 5α-reductase inhibitor alone or in combination with tamsulosin.

#### Propensity score matched analysis

We included 9553 patients (2090 dutasteride, 3445 finasteride, and 4018 tamsulosin) in the propensity score matching, and most baseline characteristics (specifically body mass index) did not differ (table 1). Duration of BPH was longer for the tamsulosin group than for the dutasteride and finasteride groups.

During a mean follow-up time of 5.7 (SD 3.2) years (6.5 (3.1), 5.9 (5.8), and 5.1 (4.7) for dutasteride, finasteride, and tamsulosin, respectively), 105, 127, and 144 new onset type 2 diabetes events were recorded. The event rate per 10 000 person years was 77.2 (95% confidence interval 62.5 to 91.9) for dutasteride, 71.3 (59.7 to 82.9) for finasteride, and 62.0 (51.2 to 72.7) for tamsulosin. The risk of type 2 diabetes was greater in patients receiving dutasteride than in those receiving tamsulosin (increased cumulative incidence; fig 2), with a point estimate hazard ratio of 1.34 (95% confidence interval 1.02 to 1.75). After propensity score matching, the rate of type 2 diabetes in the finasteride cohort no longer differed from the rate for tamsulosin (1.22; 0.95 to 1.57; table 2). However, there was an increased risk of type 2 diabetes for finasteride in the total cohorts. Again, the risk did not differ between dutasteride and finasteride when prescribed alone (1.08; 0.83 to 1.40) or when we included patients receiving these drugs in combination with tamsulosin (1.04; 0.82 to 1.31).

### Replication cohort: NHIRD

**Fig 3:**
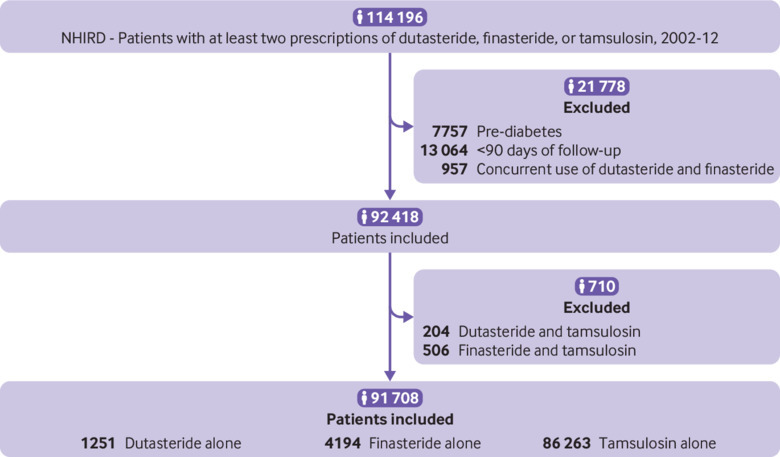
Flow chart showing Taiwanese National Health Insurance Research Database (NHIRD) study cohorts

Figure 3 shows the cohort selection for the NHIRD, and table 3 (prescribed single drugs) and supplementary table S3 (total cohorts receiving single drugs or combination treatment with tamsulosin) present the baseline characteristics. Taiwanese patients were younger than UK patients and took fewer drugs for cardiovascular indications, but more oral corticosteroids. Similarly to the UK patients, Taiwanese patients receiving tamsulosin were slightly younger than those receiving dutasteride or finasteride. More patients had dyslipidaemia in the dutasteride group.

**Table 3:** Baseline characteristics for Taiwanese National Health Insurance Research Database cohorts receiving single drugs before and after propensity matching. Values are means (standard deviations) unless stated otherwise

Characteristics	Before propensity score matching					After propensity score matching			
	Dutasteride (n=1251)	Finasteride (n=4194)	Tamsulosin (n=86 263)	P value		Dutasteride (n=1251)	Finasteride (n=2445)	Tamsulosin (n=2502)	P value
Age (years)	68.5 (10.1)	68.3 (10.5)	65.7 (11.6)	<0.01	68.5 (10.1)	68.4 (10.7)	68.3 (10.4)	0.07
Body mass index category*:								
<18.5	NA	NA	NA	NA	NA	NA	NA	NA
18.5-25	NA	NA	NA	NA	NA	NA	NA	NA
25-30	NA	NA	NA	NA	NA	NA	NA	NA
>30	NA	NA	NA	NA	NA	NA	NA	NA
Smoking status*:								
Yes	NA	NA	NA	NA	NA	NA	NA	NA
No	NA	NA	NA	NA	NA	NA	NA	NA
Former	NA	NA	NA	NA	NA	NA	NA	NA
Physical activity*:								
Inactive	NA	NA	NA	NA	NA	NA	NA	NA
Gentle	NA	NA	NA	NA	NA	NA	NA	NA
Moderate	NA	NA	NA	NA	NA	NA	NA	NA
Vigorous	NA	NA	NA	NA	NA	NA	NA	NA
Alcohol consumption*:								
Yes	NA	NA	NA	NA	NA	NA	NA	NA
No	NA	NA	NA	NA	NA	NA	NA	NA
Former	NA	NA	NA	NA	NA	NA	NA	NA
Median (interquartile range) duration of BPH (days)*	30 (0-250)	28 (0-212)	21 (39-724)	<0.01	30 (0-250)	28 (0-161)	44 (0-372)	<0.01
Disease history (No (%))								
COPD	106 (8.5)	415 (9.9)	7491 (8.7)	<0.01	106 (8.5)	210 (8.6)	210 (8.4)	0.66
Dyslipidaemia	284 (22.7)	630 (15)	14 609 (16.9)	<0.01	284 (22.7)	545 (22.3)	588 (23.5)	0.61
Hypertension	631 (50.4)	1829 (43.6)	36 653 (42.5)	<0.01	631 (50.4)	1269 (51.9)	1231 (49.2)	0.11
Other drug use (No (%))†:								
β blocker	328 (26.2)	1024 (24.4)	20 868 (24.2)	<0.01	328 (26.2)	645 (26.4)	668 (26.7)	0.71
Statin	226 (18.1)	407 (9.7)	968 (11.2)	<0.01	226 (18.1)	369 (15.1)	503 (20.1)	0.09
ACE inhibitor	300 (24.0)	956 (22.8)	17 252 (20.0)	<0.01	300 (24.0)	619 (25.3)	535 (21.4)	0.12
ARB	131 (10.5)	481 (11.5)	8799 (10.2)	<0.01	131 (10.5)	276 (11.3)	270 (10.8)	0.64
Diuretic	168 (13.5)	620 (14.8)	12 718 (14.7)	<0.01	168 (13.5)	296 (12.1)	353 (14.1)	0.43
Oral corticosteroid	342 (27.3)	1095 (26.1)	24 177 (28.0)	<0.01	342 (27.3)	719 (29.4)	681 (27.2)	0.14
No of outpatient visits‡	11.6 (8.8)	9.9 (9.6)	10.3 (9.2)	<0.01	11.6 (8.8)	10.2 (10.2)	11.2 (10.4)	<0.01

ACE=angiotensin converting enzyme; ARB=angiotensin receptor blocker; BMI=body mass index; BPH=benign prostatic hyperplasia; COPD=chronic obstructive pulmonary disease; NA=not available.

* Excluding missing data.

† In previous year.

‡ Three months before or three months after index date.

#### Analysis of main Taiwanese cohort

We recorded 1028 new onset type 2 diabetes events (21, 68, and 939 for the dutasteride, finasteride, and tamsulosin, respectively) during a mean follow-up time of 3.1 (SD 4.5) years (2.2 (3.2), 3.4 (4.7), and 2.9 (4.5) for dutasteride, finasteride, and tamsulosin). The event rate per 10 000 person years was 152.8 (95% confidence interval 144.5 to 161.5) for dutasteride and 109.1 (105.9 to 112.5) for finasteride compared with 74.7 (74.2 to 75.2) for tamsulosin. The event rates for the total cohorts were similar (155.2, 142.1 to 169.1 for dutasteride combined with tamsulosin; 111.3, 97.1 to 127.7 for finasteride combined with tamsulosin).

**Fig 4:**
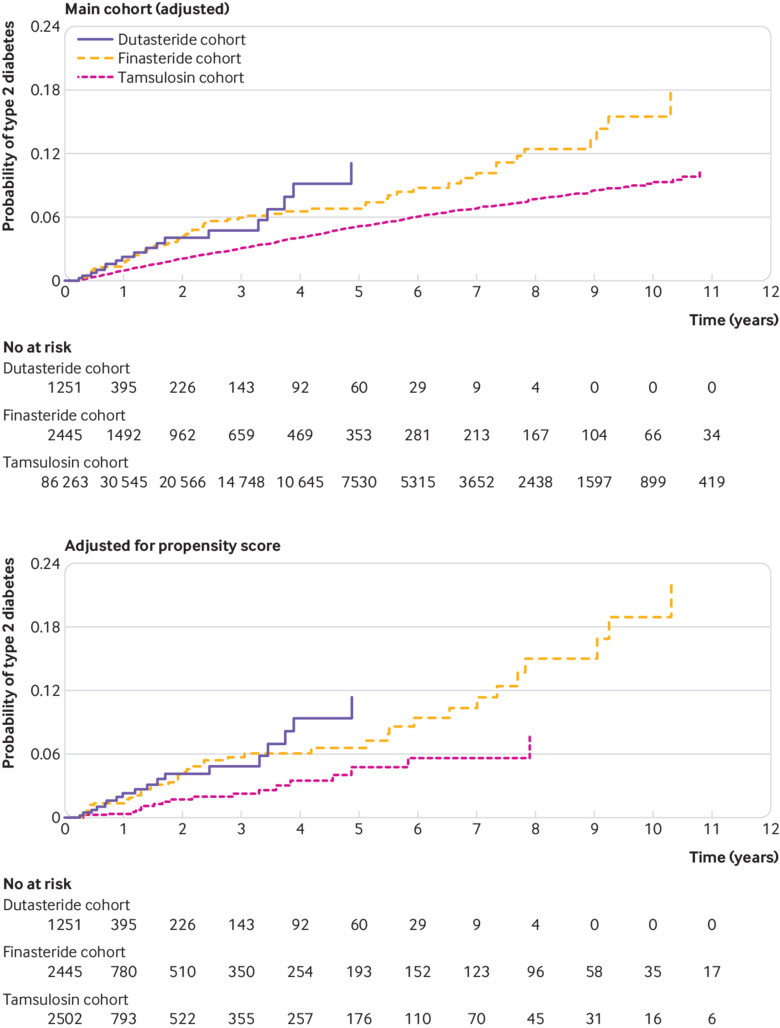
Cumulative incidence of type 2 diabetes mellitus in Taiwanese National Health Insurance Research Database cohort over study period: main cohort (adjusted), and adjusted for propensity score

The risk of developing type 2 diabetes (table 2) was higher for patients receiving dutasteride or finasteride compared with tamsulosin (adjusted hazard ratio 1.34, 95% confidence interval 1.17 to 1.54; and 1.49, 1.38 to 1.61, respectively), but the risk for dutasteride and finasteride did not differ (0.90, 0.77 to 1.06). Figure 4 shows the increased cumulative incidence of type 2 diabetes. Results were similar in the total cohorts when we included patients receiving combination treatment (table 2).

#### Propensity score matched analysis

We included 6198 patients (1251 dutasteride, 2445 finasteride, and 2502 tamsulosin) in the propensity score matching. Baseline characteristics were similar among cohorts (table 3).

The mean follow-up times were 2.9 (SD 3.4) years (2.2 (3.2), 3.2 (3.5), and 2.9 (3.3) for dutasteride, finasteride, and tamsulosin, respectively). The associated event rates per 10 000 person years for type 2 diabetes were 152.8 (95% confidence interval 144.5 to 161.5), 111.4 (107.1 to 115.8), and 79.7 (76.6 to 82.9), with 21, 35, and 25 new onset events recorded. The risk of type 2 diabetes in patients receiving dutasteride and finasteride was higher than in those receiving tamsulosin: hazard ratio 1.18 (95% confidence interval 1.00 to 1.40) and 1.61 (1.46 to 1.80), respectively; however, the risk did not differ between dutasteride and finasteride (0.94, 0.80 to 1.11; table 2). Figure 4 shows the increased cumulative incidence of type 2 diabetes. These findings were corroborated by the results for the total cohorts, which also included patients receiving combination treatments (table 2).

## Discussion

Previous short term studies suggest that dutasteride but not finasteride might increase the risk of type 2 diabetes by impairing insulin sensitivity and increasing steatosis.^8^ ^9^ ^13^ ^14^ ^19^ Our pharmacoepidemiological analyses show that the incidence of type 2 diabetes was increased in patients with BPH receiving dutasteride compared with tamsulosin, but an increased risk was also seen in patients receiving finasteride. In the UK, for every 10 000 person years of treatment, type 2 diabetes occurs in 76 patients treated with dutasteride or finasteride, but only in 60 patients treated with tamsulosin. These data are comparable to the increased incidence of type 2 diabetes with statin use.^2^ The risk we identified is modest, but consistent between the two study populations. We performed a sensitivity analysis by propensity score matching for differences in baseline variables, including body mass index and statin use, and the increased risk of developing type 2 diabetes remained. Our findings were replicated in the Taiwanese cohort: we recorded 78 and 35 extra events for every 10 000 person years of treatment for dutasteride and finasteride, respectively, compared with tamsulosin, against an overall higher incidence of type 2 diabetes in the Taiwanese population. We observed this increased risk whether dutasteride or finasteride was prescribed alone or combined with tamsulosin.

### Strengths and weaknesses of this study

A major strength of this study was the cohort design. We used two large healthcare databases with different ethnic populations and made adjustments for known confounders. Our findings reflect usual healthcare practice in the UK and can be generalised to other populations. Nonetheless, differences in practice were apparent between the two countries. The healthcare system in Taiwan does not have primary and secondary care divisions and has less emphasis on health screening and preventive treatments, such as statins. Overall type 2 diabetes incident rates were higher in Taiwan than in the UK, with younger patients affected. This finding is consistent with Asian populations having higher incident rates of type 2 diabetes than European populations.^20^ ^21^ ^22^ The Taiwanese database does not contain data for body mass index, smoking status, and alcohol consumption, and we could not control for these potential biases. Body mass index is a potential confounding risk factor for type 2 diabetes, but the comparable risk for both 5α-reductase inhibitors persisted in the CPRD cohort after adjustment and after effective matching by propensity score. Moreover, we conducted a further survival analysis with a time dependent variable of body mass index, and the results remain similar (supplementary table S4). In the NHIRD there is also a lack of detailed information on socioeconomic factors, lifestyle behaviours, imaging, and biochemical data. The follow-up period was shorter in Taiwan and could reflect differences in prescribing practices or the healthcare setting. We used the well established method of grouping all missing data into one category in the final model, rather than multiple imputation; multiple imputation makes the assumption that data are missing at random, which is often not the case with medical data.

The study had some weaknesses. Misclassification of coding for the exposures, outcomes, and covariates might exist, although previous evidence attests to the accuracy of CPRD^16^ and NHIRD data.^17^ ^23^ ^24^ Assessments were made on the basis of prescriptions, and adherence to drugs was not monitored. We calculated the duration of disease before the index date from the first record of BPH; however, in some patients this coincided with the start of treatment (defined as duration of zero), and it is possible the disease was present before this time point. A dose-response relation could not be assessed because 5α-reductase inhibitors are only used at a single dose. We censored patients if they switched between dutasteride and finasteride but not if they stopped treatment; however, this would only underestimate the risk. The present study is observational, non-randomised, and unblinded, therefore we could not control for unmeasured confounding factors and biases using propensity score matching. General practice prescribing preferences were not available; this information could be used in an instrumental variable analysis, a technique that deals with unmeasured confounders. However, we anticipated that the impact of unmeasured cofounders would be small, in line with our previous findings for the Medicines Monitoring Unit database.^25^

The power in the CPRD study was constrained by the number of patients receiving dutasteride. Finasteride has been “off patent” for longer, whereas generic dutasteride has only been available since 2014. However, given the substantial overlap in the risks between finasteride and dutasteride, and replication of the findings in Taiwan, a difference in risk would probably not be uncovered with larger numbers. The characteristics of the most appropriate control population in the setting of type 2 diabetes in BPH are debatable, and untreated patients are not available for study across a suitable timescale. There are inherent differences in disease severity in patients receiving tamsulosin compared with those receiving 5α-reductase inhibitors; 5α-reductase inhibitors are prescribed in patients with enlarged prostates and tamsulosin is favoured as first line treatment for lower urinary tract symptoms. These differences in indications could differentiate between two patient populations that have varying susceptibilities to metabolic disease.

The most probable confounders are metabolic risk factors, and we sought to address these factors as far as possible through propensity score matching. Scoring with body mass index in the CPRD data allowed matching of obesity at the index date, and this did not change the outcome. However, longer duration of obesity before the study period might have resulted in an enlarged prostate and thus a greater likelihood of prescription of 5α-reductase inhibitors. We further analysed data with a time dependent variable of body mass index, and the results showed little difference. The propensity score matched analysis included only a quarter of the patient cohort because of predictable difficulties matching baseline variables in these cohorts with high precision. Although the possibilities of an unknown mechanism and reverse causality remain, the patient groups on 5α-reductase inhibitors are at an increased risk of type 2 diabetes.

An alternative hypothesis could be that tamsulosin might protect patients from developing type 2 diabetes as opposed to 5α-reductase inhibitors accelerating the process. However, because the diagnosis of BPH was made on presentation of symptoms and owing to the effectiveness of treatment, it was not possible to study patients without intervention over this timescale. Reassuringly, the rates of type 2 diabetes in patients receiving tamsulosin (approximately 60 per 10 000 person years) were comparable to those in the general population (42-64 per 10 000 patients^26^). Importantly, inclusion of patients receiving tamsulosin combined with a 5α-reductase inhibitor revealed the same increased risks of type 2 diabetes as patients receiving a 5α-reductase inhibitor alone. However, numbers of patients using combination treatments were low, preventing a meaningful analysis of these groups.

In an additional analysis, we compared the rates of new incident type 2 diabetes with patients receiving surgical treatment: transurethral resection of the prostate. We found some evidence in the NHIRD cohort that patients receiving tamsulosin were less likely to develop type 2 diabetes than patients undergoing surgery, but this was not evident in the CPRD cohort (supplementary tables S5 and S6). However, these patient groups were poorly matched, even after propensity scoring. We conclude that there is a possibility tamsulosin offers some protection against type 2 diabetes, but there is an adverse effect of 5α-reductase inhibitors. A randomised controlled trial is needed to study this hypothesis further.

### Strengths and weaknesses in relation to other studies

In phase III trials of dutasteride, no evidence was found of increased blood glucose levels,^27^ ^28^ but these studies were only for two years and were limited in statistical power. In this study, we observed the increased risk with 5α-reductase inhibitors after approximately three years of treatment, which is consistent with a previous report of the slow progression to type 2 diabetes with obesity and changes in fasting glucose.^12^ The only previous longer term observational studies were focused on heart failure and had inconsistent results.^29^ ^30^ A previous pharmacoepidemiological study conducted over five years suggested a lower risk of type 2 diabetes, but had smaller numbers of patients, a less well characterised control group, and importantly, did not include adjustment for body mass index.^15^

Based on previous experimental medicine studies, we anticipated an increased risk of type 2 diabetes only in the dutasteride cohort; the greater risk of type 2 diabetes in patients receiving finasteride contrasts with its lack of measurable effect on metabolism after three months of treatment.^8^ However, although the point measures of the hazard ratios suggest an increased risk of approximately 30% in the UK population for dutasteride and finasteride, the relation was weaker with finasteride.

In rodents, disruption of 5αR1 but not 5αR2 causes insulin resistance, fatty liver, and susceptibility to liver fibrosis.^13^ ^14^ However, in rodent liver only 5αR1 is expressed, whereas in human liver both isozymes of 5α-reductases are present.^8^ Urinary steroid profiling in humans showed robust reductions in 5α reduced steroid metabolites with finasteride and dutasteride,^8^ although dutasteride lowers circulating dihydrotestosterone to a greater extent than finasteride (approximately 90% *v* 70%).^31^ Thus, it is plausible that both drugs affect liver metabolism. However, changes in insulin sensitivity with dutasteride can be detected sooner using sensitive euglycemic hyperinsulinemic clamps because of its greater potency, while the more modest biochemical effect of finasteride has an accumulated risk over time.

### Meaning of the study

Tamsulosin is recommended as first line treatment for patients with symptomatic BPH.^10^ 5α-reductase inhibitors are recommended especially in patients with larger prostates, but currently no guidance is available about choosing dutasteride or finasteride. In this study, the risk of type 2 diabetes was similar for dutasteride and finasteride. Patients receiving these drugs are older and more susceptible to metabolic disease. Therefore, it will be important to consider the risk of development or exacerbation of type 2 diabetes when prescribing these drugs and to apply suitable monitoring strategies.

This study should alert clinicians that patients starting 5α-reductase inhibitor treatment might benefit from early lifestyle advice and monitoring of type 2 diabetes.

### Unanswered questions and future research

Why are patients receiving 5α-reductase inhibitors at increased risk of type 2 diabetes? 5α-reductases are highly expressed in the liver, but also in other tissues critical for insulin sensitivity—for example adipose tissue and skeletal muscle. The enzymes metabolise a range of steroids, including testosterone, cortisol, progesterone, and aldosterone. The increased susceptibility to type 2 diabetes might reflect changes in these hormones, most plausibly androgens or glucocorticoids. 5α-dihydrotestosterone is a more active androgen than testosterone, so inhibiting its formation with dutasteride or finasteride^32^ could induce features of androgen deficiency, which include insulin resistance. Low circulating levels of testosterone are associated with an increased risk of type 2 diabetes^33^ in men, a relation also obvious after androgen deprivation therapy in prostate cancer.^34^ Interestingly the patients we studied will have low 5α-dihydrotestosterone but their levels of testosterone are possibly higher; therefore, they would only be androgen deficient at a localised tissue level where 5α-reductases are expressed, including liver and adipose.^8^ Importantly castration in rodent models did not protect against the adverse metabolic effects of 5α-reductase inhibitors,^13^ which suggests other factors are at play. Prevention of inactivation of cortisol by 5α-reductases could lead to accumulation of glucocorticoid in metabolic tissues, again promoting insulin resistance.

The increased risk of type 2 diabetes has been studied up to 11 years and might continue to rise; therefore, patients would require longer follow-up, particularly considering treatment could be lifelong. Further investigation is now required to uncover whether these patients are also more at risk of diabetic complications. Finally, further studies could explore the unmeasured confounders by conducting an instrumental variable analysis, with the instrumental variable carefully chosen and validated.

### Conclusion

Men using steroid 5α-reductase inhibitors for BPH appear to be at a modest increased risk of developing type 2 diabetes. Caveats exist about biases and confounders within population cohort studies. However, in the light of our findings, the decision to prescribe 5α-reductase inhibitors for men with metabolic disease must be considered carefully in the context of other risk factors for type 2 diabetes. In addition, monitoring of fasting glucose might be advisable.

**What is already known on this topic**

Several commonly used drugs increase the risk of type 2 diabetes mellitus
Recent short term experimental medicine studies of dutasteride (a steroid 5α-reductase inhibitor used to treat prostatic disease) have shown that it induces insulin resistance and fatty liver
Men who regularly receive dutasteride might be at increased risk of type 2 diabetes compared with those who use alternative treatments

**What this study adds**

The risk of type 2 diabetes was increased by approximately 30% over 11 years in men with benign prostatic hyperplasia who received one of the 5α-reductase inhibitors on the market, finasteride or dutasteride compared with tamsulosin
The decision to prescribe steroid 5α-reductase inhibitors for men with metabolic disease should be made in the context of other risk factors for type 2 diabetes; additional monitoring of blood glucose might be advisable

## Web extra

Extra material supplied by authors

## Notes

Ethical approval: The study protocol was approved by the independent scientific advisory committee for UK Medicines and Healthcare Products Regulatory Agency database research (protocol No 13_189) and the institutional review board, National Cheng Kung University Hospital (protocol No -/B-EX-105-023).

Data sharing: No additional data are available.

Transparency: The lead author (the manuscript’s guarantor) affirms that the manuscript is an honest, accurate, and transparent account of the study bring reported; that no important aspects of the study have been omitted; and that any discrepancies from the study as planned (and, if relevant, registered) have been explained.

## References

[1] Gress TD Nieto FJ Sharar E Wofford MR Brancati FL Hypertension and antihypertensive therapy as risk factors for type 2 diabetes mellitus. Atherosclerosis Risk in Communities Study. N Engl J Med 2000;342:905-12. 10.1056/NEJM200003303421301 10738048

[2] Sattar N Preiss D Murray HM Statins and risk of incident diabetes: a collaborative meta-analysis of randomised statin trials. Lancet 2010;375:735-42. 10.1016/S0140-6736(09)61965-6 20167359

[3] Preiss D Seshasai SRK Welsh P Risk of incident diabetes with intensive-dose compared with moderate-dose statin therapy: a meta-analysis. JAMA 2011;305:2556-64. 10.1001/jama.2011.860 21693744

[4] Holt RIG Peveler RC Association between antipsychotic drugs and diabetes. Diabetes Obes Metab 2006;8:125-35. 10.1111/j.1463-1326.2005.00495.x 16448516

[5] Brown TT Cole SR Li X Antiretroviral therapy and the prevalence and incidence of diabetes mellitus in the multicenter AIDS cohort study. Arch Intern Med 2005;165:1179-84. 10.1001/archinte.165.10.1179 15911733

[6] Heisel O Heisel R Balshaw R Keown P New onset diabetes mellitus in patients receiving calcineurin inhibitors: a systematic review and meta-analysis. Am J Transplant 2004;4:583-95. 10.1046/j.1600-6143.2003.00372.x 15023151

[7] Gurwitz JH Bohn RL Glynn RJ Monane M Mogun H Avorn J Glucocorticoids and the risk for initiation of hypoglycemic therapy. Arch Intern Med 1994;154:97-101. 10.1001/archinte.1994.00420010131015 8267494

[8] Upreti R Hughes KA Livingstone DE 5α-reductase type 1 modulates insulin sensitivity in men. J Clin Endocrinol Metab 2014;99:E1397-406. 10.1210/jc.2014-1395 24823464 PMC4207930

[9] Hazlehurst JM Oprescu AI Nikolaou N Dual-5α-Reductase Inhibition Promotes Hepatic Lipid Accumulation in Man. J Clin Endocrinol Metab 2016;101:103-13. 10.1210/jc.2015-2928 26574953 PMC4701851

[10] Services UDoHaH. *Guideline on the management of benign prostatic hyperplasia (BPH)* *.* 2010 NGC-8255.

[11] Bechis SK Otsetov AG Ge R Olumi AF Personalized medicine for the management of benign prostatic hyperplasia. J Urol 2014;192:16-23. 10.1016/j.juro.2014.01.114 24582540 PMC4143483

[12] Traish A Haider KS Doros G Haider A Long-term dutasteride therapy in men with benign prostatic hyperplasia alters glucose and lipid profiles and increases severity of erectile dysfunction. Horm Mol Biol Clin Investig 2017;30:1-16. 10.1515/hmbci-2017-0015 28632494

[13] Livingstone DE Barat P Di Rollo EM 5α-Reductase type 1 deficiency or inhibition predisposes to insulin resistance, hepatic steatosis, and liver fibrosis in rodents. Diabetes 2015;64:447-58. 10.2337/db14-0249 25239636

[14] Dowman JK Hopkins LJ Reynolds GM Loss of 5α-reductase type 1 accelerates the development of hepatic steatosis but protects against hepatocellular carcinoma in male mice. Endocrinology 2013;154:4536-47. 10.1210/en.2013-1592 24080367 PMC4192287

[15] Lee SS Yang YW Tsai TH 5-alpha-reductase inhibitors and the risk of diabetes mellitus: A nationwide population-based study. Prostate 2016;76:41-7. 10.1002/pros.23097 26390988

[16] Herrett E Thomas SL Schoonen WM Smeeth L Hall AJ Validation and validity of diagnoses in the General Practice Research Database: a systematic review. Br J Clin Pharmacol 2010;69:4-14. 10.1111/j.1365-2125.2009.03537.x 20078607 PMC2805870

[17] Cheng CL Kao YH Lin SJ Lee CH Lai ML Validation of the National Health Insurance Research Database with ischemic stroke cases in Taiwan. Pharmacoepidemiol Drug Saf 2011;20:236-42. 10.1002/pds.2087 21351304

[18] Lai EC Hsieh CY Wong MB Comparative risk of oral ulcerations among antipsychotics users - population-based retrospective cohort study. Pharmacoepidemiol Drug Saf 2016;25:123-32. 10.1002/pds.3903 26549190

[19] Livingstone DE Di Rollo EM Mak TC Sooy K Walker BR Andrew R Metabolic dysfunction in female mice with disruption of 5α-reductase 1. J Endocrinol 2017;232:29-36. 10.1530/JOE-16-0125 27647861 PMC5118938

[20] Chan JCN Malik V Jia W Diabetes in Asia: epidemiology, risk factors, and pathophysiology. JAMA 2009;301:2129-40. 10.1001/jama.2009.726 19470990

[21] Organisation WH Global Reports on Diabetes. WHO, 2016.

[22] Bank TW. Diabetes prevalence (% of population ages 20 to 79). 2018; https://data.worldbank.org/indicator/SH.STA.DIAB.ZS. Accessed 18/11/2018, 2018.

[23] Chen CC Chen LS Yen MF Chen HH Liou HH Geographic variation in the age- and gender-specific prevalence and incidence of epilepsy: analysis of Taiwanese National Health Insurance-based data. Epilepsia 2012;53:283-90. 10.1111/j.1528-1167.2011.03332.x 22126307

[24] Wu CY Chan FK Wu MS Histamine2-receptor antagonists are an alternative to proton pump inhibitor in patients receiving clopidogrel. Gastroenterology 2010;139:1165-71. 10.1053/j.gastro.2010.06.067 20600012

[25] Wei L MacDonald TM Walker BR Taking glucocorticoids by prescription is associated with subsequent cardiovascular disease. Ann Intern Med 2004;141:764-70. 10.7326/0003-4819-141-10-200411160-00007 15545676

[26] Zghebi SS Steinke DT Carr MJ Rutter MK Emsley RA Ashcroft DM Examining trends in type 2 diabetes incidence, prevalence and mortality in the UK between 2004 and 2014. Diabetes Obes Metab 2017;19:1537-45. 28387052 10.1111/dom.12964

[27] Schulman C Pommerville P Höfner K Wachs B Long-term therapy with the dual 5alpha-reductase inhibitor dutasteride is well tolerated in men with symptomatic benign prostatic hyperplasia. BJU Int 2006;97:73-9, discussion 79-80. 10.1111/j.1464-410X.2005.05909.x 16336332

[28] Andriole GL Kirby R Safety and tolerability of the dual 5alpha-reductase inhibitor dutasteride in the treatment of benign prostatic hyperplasia. Eur Urol 2003;44:82-8. 10.1016/S0302-2838(03)00198-2 12814679

[29] Andriole GL Bostwick DG Brawley OW REDUCE Study Group Effect of dutasteride on the risk of prostate cancer. N Engl J Med 2010;362:1192-202. 10.1056/NEJMoa0908127 20357281

[30] Loke YK Ho R Smith M Systematic review evaluating cardiovascular events of the 5-alpha reductase inhibitor - Dutasteride. J Clin Pharm Ther 2013;38:405-15. 10.1111/jcpt.12080 23815285

[31] Gisleskog PO Hermann d, Hammarlund-Udenaes M, Karlsson MO. A model for the turnover of dihydrotestosterone in the presence of the irreversible 5α-reductase inhibitors GII98745 and finasteride. Clin Pharmacol Ther 1998;64:636-47. 10.1016/S0009-9236(98)90054-6 9871428

[32] Clark RV Hermann DJ Cunningham GR Wilson TH Morrill BB Hobbs S Marked suppression of dihydrotestosterone in men with benign prostatic hyperplasia by dutasteride, a dual 5α-reductase inhibitor. J Clin Endocrinol Metab 2004;89:2179-84. 10.1210/jc.2003-030330 15126539

[33] Hackett G Type 2 Diabetes and testosterone therapy. World J Mens Health 2019;37:31-44. 10.5534/wjmh.180027. 30079639 PMC6305869

[34] Jhan JH Yeh HC Chang YH New-onset diabetes after androgen-deprivation therapy for prostate cancer: A nationwide propensity score-matched four-year longitudinal cohort study. J Diabetes Complications 2018;32:688-92. 10.1016/j.jdiacomp.2018.03.007 29909141

