# Supplementary material for: Incidence of type 2 diabetes mellitus in men receiving steroid 5α-reductase inhibitors: population based cohort study
Source: BMJ. 2019 Apr 10;365:l1204. doi: 10.1136/bmj.l1204 (PMC6456811; doi:10.1136/bmj.l1204)
Supplement: Supplementary file 1 — Supplementary information: additional tables, S1-S6 [file weil046562.ww1.pdf]

On-line Only Supplements

1) Main analysis: on-line only Tables S1-4
2) Assessment of Confounding but Tamsulosin with online-only Tables S5-S6.

Supplementary Table S1A: Read codes for Benign Prostatic Hyperplasia and Transurethral resection of the prostate

Read codes are the standard clinical terminology system used in General Practice in the United Kingdom.

**Benign prostatic hypertrophy**	
**Read Code**	**Read Term**
K20..15	BPH - benign prostatic hypertrophy
K20..00	Benign prostatic hypertrophy
K200.00	Prostatic hyperplasia unspecified
K20z.00	Prostatic hyperplasia NOS
**Transurethral resection of the prostate (TURPS)**	
**Read Code**	**Read Term**
7B39000	Transurethral prostatectomy
7B3C500	Transurethral biopsy prostate
7B3A400	Transurethral incision of prostate
7B3B900	Endoscopic transurethral electrovaporisation of prostate

Supplementary Table S1B: Read codes for Type 2 Diabetes Mellitus in CPRD database

Read Code	Read Term
ZV65312	[V]Dietary counselling in diabetes mellitus
Cyu2.00	[X]Diabetes mellitus
Cyu2000	[X]Other specified diabetes mellitus
F171100	Autonomic neuropathy due to diabetes
C104z00	Diabetes mellitis with nephropathy NOS
C10..00	Diabetes mellitus
C10C.00	Diabetes mellitus autosomal dominant
C10D.00	Diabetes mellitus autosomal dominant type 2
C102z00	Diabetes mellitus NOS with hyperosmolar coma
C101z00	Diabetes mellitus NOS with ketoacidosis
C103z00	Diabetes mellitus NOS with ketoacidotic coma
C106z00	Diabetes mellitus NOS with neurological manifestation
C100z00	Diabetes mellitus NOS with no mention of complication
C105z00	Diabetes mellitus NOS with ophthalmic manifestation
C10yz00	Diabetes mellitus NOS with other specified manifestation
C107z00	Diabetes mellitus NOS with peripheral circulatory disorder
C10zz00	Diabetes mellitus NOS with unspecified complication
C107.11	Diabetes mellitus with gangrene
C102.00	Diabetes mellitus with hyperosmolar coma
C101.00	Diabetes mellitus with ketoacidosis
C103.00	Diabetes mellitus with ketoacidotic coma
C106.00	Diabetes mellitus with neurological manifestation
C106.12	Diabetes mellitus with neuropathy
C100.00	Diabetes mellitus with no mention of complication
C105.00	Diabetes mellitus with ophthalmic manifestation
C10y.00	Diabetes mellitus with other specified manifestation
C107.00	Diabetes mellitus with peripheral circulatory disorder
C106.13	Diabetes mellitus with polyneuropathy
C104.00	Diabetes mellitus with renal manifestation

C10z.00	Diabetes mellitus with unspecified complication
C106100	Diabetes mellitus, adult onset, + neurological manifestation
C105100	Diabetes mellitus, adult onset, + ophthalmic manifestation
C10z100	Diabetes mellitus, adult onset, + unspecified complication
C100100	Diabetes mellitus, adult onset, no mention of complication
C102100	Diabetes mellitus, adult onset, with hyperosmolar coma
C101100	Diabetes mellitus, adult onset, with ketoacidosis
C103100	Diabetes mellitus, adult onset, with ketoacidotic coma
C104100	Diabetes mellitus, adult onset, with renal manifestation
C107200	Diabetes mellitus, adult with gangrene
C10y100	Diabetes mellitus, adult, + other specified manifestation
C107100	Diabetes mellitus, adult, + peripheral circulatory disorder
66Ao.00	Diabetes type 2 review
ZC2CA00	Dietary advice for type II diabetes
2G51000	Foot abnormality - diabetes related
2G5C.00	Foot abnormality - diabetes related
C109K00	Hyperosmolar non-ketotic state in type 2 diabetes mellitus
C10FK00	Hyperosmolar non-ketotic state in type 2 diabetes mellitus
C10A100	Malnutrition-related diabetes mellitus with ketoacidosis
K01x100	Nephrotic syndrome in diabetes mellitus
C109.11	NIDDM - Non-insulin dependent diabetes mellitus
C109E00	Non-insulin depend diabetes mellitus with diabetic cataract
C109700	Non-insulin dependent diabetes mellitus - poor control
C109.00	Non-insulin dependent diabetes mellitus
C109G00	Non-insulin dependent diabetes mellitus with arthropathy
C109500	Non-insulin dependent diabetes mellitus with gangrene
C109D00	Non-insulin dependent diabetes mellitus with hypoglyca coma
C109A00	Non-insulin dependent diabetes mellitus with mononeuropathy
C109C00	Non-insulin dependent diabetes mellitus with nephropathy
C109B00	Non-insulin dependent diabetes mellitus with polyneuropathy
C109400	Non-insulin dependent diabetes mellitus with ulcer
C109300	Non-insulin-dependent diabetes mellitus with multiple comps
C109200	Non-insulin-dependent diabetes mellitus with neuro comps
C109100	Non-insulin-dependent diabetes mellitus with ophthalm comps
C109000	Non-insulin-dependent diabetes mellitus with renal comps
C109600	Non-insulin-dependent diabetes mellitus with retinopathy
C109900	Non-insulin-dependent diabetes mellitus without complication
C103y00	Other specified diabetes mellitus with coma
C101y00	Other specified diabetes mellitus with ketoacidosis
C108y00	Other specified diabetes mellitus with multiple comps
C106y00	Other specified diabetes mellitus with neurological comps
C105y00	Other specified diabetes mellitus with ophthalmic complicatn
C10yy00	Other specified diabetes mellitus with other spec comps
C104y00	Other specified diabetes mellitus with renal complications
C10zy00	Other specified diabetes mellitus with unspecified comps
8BL2.00	Patient on maximal tolerated therapy for diabetes
ZRbH.00	Perceived control of insulin-dependent diabetes
F372.00	Polyneuropathy in diabetes
L180600	Pre-existing diabetes mellitus, non-insulin-dependent
2BBF.00	Retinal abnormality - diabetes related
C10N.00	Secondary diabetes mellitus
C10N000	Secondary diabetes mellitus without complication
C109.12	Type 2 diabetes mellitus
C10F.00	Type 2 diabetes mellitus
C109712	Type 2 diabetes mellitus - poor control
C10F700	Type 2 diabetes mellitus - poor control
C109G12	Type 2 diabetes mellitus with arthropathy
C10FG00	Type 2 diabetes mellitus with arthropathy

C109E12	Type 2 diabetes mellitus with diabetic cataract
C10FE00	Type 2 diabetes mellitus with diabetic cataract
C10FQ00	Type 2 diabetes mellitus with exudative maculopathy
C109512	Type 2 diabetes mellitus with gangrene
C10F500	Type 2 diabetes mellitus with gangrene
C10FR00	Type 2 diabetes mellitus with gastroparesis
C109D12	Type 2 diabetes mellitus with hypoglycaemic coma
C10FD00	Type 2 diabetes mellitus with hypoglycaemic coma
C10FN00	Type 2 diabetes mellitus with ketoacidosis
C10FP00	Type 2 diabetes mellitus with ketoacidotic coma
C10FA00	Type 2 diabetes mellitus with mononeuropathy
C10F300	Type 2 diabetes mellitus with multiple complications
C109C12	Type 2 diabetes mellitus with nephropathy
C10FC00	Type 2 diabetes mellitus with nephropathy
C109212	Type 2 diabetes mellitus with neurological complications
C10F200	Type 2 diabetes mellitus with neurological complications
C109H12	Type 2 diabetes mellitus with neuropathic arthropathy
C10FH00	Type 2 diabetes mellitus with neuropathic arthropathy
C109112	Type 2 diabetes mellitus with ophthalmic complications
C10F100	Type 2 diabetes mellitus with ophthalmic complications
C109F12	Type 2 diabetes mellitus with peripheral angiopathy
C10FF00	Type 2 diabetes mellitus with peripheral angiopathy
C10FM00	Type 2 diabetes mellitus with persistent microalbuminuria
C10FL00	Type 2 diabetes mellitus with persistent proteinuria
C10FB00	Type 2 diabetes mellitus with polyneuropathy
C109012	Type 2 diabetes mellitus with renal complications
C10F000	Type 2 diabetes mellitus with renal complications
C109612	Type 2 diabetes mellitus with retinopathy
C10F600	Type 2 diabetes mellitus with retinopathy
C109412	Type 2 diabetes mellitus with ulcer
C10F400	Type 2 diabetes mellitus with ulcer
C10F900	Type 2 diabetes mellitus without complication
C109.13	Type II diabetes mellitus
C10F.11	Type II diabetes mellitus
C109711	Type II diabetes mellitus - poor control
C10F711	Type II diabetes mellitus - poor control
C109G11	Type II diabetes mellitus with arthropathy
C109E11	Type II diabetes mellitus with diabetic cataract
C10FE11	Type II diabetes mellitus with diabetic cataract
C109511	Type II diabetes mellitus with gangrene
C109D11	Type II diabetes mellitus with hypoglycaemic coma
C109A11	Type II diabetes mellitus with mononeuropathy
C10F311	Type II diabetes mellitus with multiple complications
C109C11	Type II diabetes mellitus with nephropathy
C109211	Type II diabetes mellitus with neurological complications
C109H11	Type II diabetes mellitus with neuropathic arthropathy
C109111	Type II diabetes mellitus with ophthalmic complications
C109F11	Type II diabetes mellitus with peripheral angiopathy
C10FM11	Type II diabetes mellitus with persistent microalbuminuria
C10FL11	Type II diabetes mellitus with persistent proteinuria
C109B11	Type II diabetes mellitus with polyneuropathy
C10FB11	Type II diabetes mellitus with polyneuropathy
C109011	Type II diabetes mellitus with renal complications
C10F011	Type II diabetes mellitus with renal complications
C109611	Type II diabetes mellitus with retinopathy
C10F611	Type II diabetes mellitus with retinopathy
C109411	Type II diabetes mellitus with ulcer
C10F411	Type II diabetes mellitus with ulcer

C10F911	Type II diabetes mellitus without complication
C108z00	Unspecified diabetes mellitus with multiple complications
66AJ.11	Unstable diabetes

Supplementary Table S2: Baseline characteristics for total cohorts, receiving single or combination therapies (UK CPRD database)

	Dutasteride ± tamsulosin N=8830	Finasteride ± tamsulosin N=33396	Tamsulosin N=16270	P value
Age (y)	71.8 (9.8)	72.2 (10.8)	69.1 (10.3)	<0.01
BMI category* (kg/m^2)				0.01
1 (<18.5)	47 (0.6)	206 (0.7)	91 (0.6)	
2 (18.5-25)	2613 (34.7)	9815 (34.9)	4794 (33.6)	
3 (25-30)	3541 (47.1)	12953 (46.1)	6846 (48.1)	
4 (>30)	1321 (17.6)	5135 (18.3)	2518 (17.7)	
Smoking status*				<0.01
1 – Yes	917 (10.8)	3540 (11.2)	1859 (11.8)	
2 – No	4058 (47.7)	14511 (45.9)	7485(47.4)	
3 – Ex	3538 (41.6)	13541 (42.9)	6458 (40.9)	
Physical activity*				<0.01
1 – Inactive	406 (9.8)	1442 (9.7)	699 (9.6)	
2 – Gentle	1259 (36.9)	5765 (38.9)	2636 (34.1)	
3 – Moderate	1989 (48.0)	6894 (46.6)	39.7 (50.7)	
4 – Vigorous	219 (5.3)	706 (4.8)	473 (6.1)	
Alcohol consumption*				<0.01
1 – Yes	6339 (82.1)	23720 (82.4)	12233 (84.4)	
2 – No	1043 (14.8)	4145 (14.4)	1850 (12.8)	
3 – Ex	238 (3.1)	914 (3.2)	416 (2.9)	
Duration of BPH (days) median (IQR)	15 (0-1292)	13 (0-1075)	56 (27-892)	<0.01
Disease history				
COPD	679 (7.7)	2818 (8.4)	1169 (7.2)	<0.01
Dyslipidaemia	1349 (15.3)	4723 (14.1)	2711 (16.7)	<0.01
Hypertension	3015 (34.1)	12053 (36.1)	5241 (32.2)	<0.01
Beta blockers	2312 (26.2)	8225 (24.6)	3471 (21.3)	<0.01
Statins	3496 (40.0)	12745 (38.2)	5871 (369.1)	<0.01
ACEs	2411 (27.3)	9350 (28.0)	3844 (23.6)	<0.01
ARBs	891 (10.1)	3038 (9.2)	1359 (8.4)	<0.01
Diuretics	2597 (29.4)	10026 (30.0)	3669 (22.6)	<0.01
Oral corticosteroids	954 (10.8)	53384 (10.1)	1497 (9.2)	<0.01
Number of GP contacts	10.6 (2.2)	10.2 (2.4)	10.0 (2.4)	<0.01

Data are numbers (%) of subjects unless otherwise stated.

BMI = Body Mass Index; BPH = Benign Prostatic Hyperplasia; COPD = Chronic Obstructive Pulmonary Disease; ACEs = Angiotensin converting enzyme inhibitors; ARBs = Angiotensin receptor blocker; GP = General Practitioner; OPD = Out-patient Department. Data show mean (standard deviation; SD) unless otherwise stated. IQR = Interquartile range. * Excluding missing data.

Supplementary Table S3: Baseline characteristics for total cohorts receiving single or combination therapies (NHIRD database)

	**Dutasteride ± tamsulosin N=1455**	**Finasteride ± tamsulosin N=4700**	**Tamsulosin N=86263**	**P value**
Age (y)	68.7 (10.7)	68.5 (10.9)	65.7 (11.6)	<0.01
BMI category* (kg/m^2)				
1 (<18.5)	NA	NA	NA	NA
2 (18.5-25)	NA	NA	NA	NA
3 (25-30)	NA	NA	NA	NA
4 (>30)	NA	NA	NA	NA
Smoking status*				
1 – Yes	NA	NA	NA	NA
2 – No	NA	NA	NA	NA
3 – Ex	NA	NA	NA	NA
Physical activity*				
1 – Inactive	NA	NA	NA	NA
2 – Gentle	NA	NA	NA	NA
3 – Moderate	NA	NA	NA	NA
4 – Vigorous	NA	NA	NA	NA
Alcohol consumption*				
1 – Yes	NA	NA	NA	NA
2 – No	NA	NA	NA	NA
3 – Ex	NA	NA	NA	NA
Duration of BPH (days) median (IQR)	29 (0-286)	27 (0-240)	21 (39-724)	<0.01
Disease history				
COPD	123 (8.5)	455 (9.7)	7491 (8.7)	<0.01
Dyslipidaemia	329 (22.6)	692 (14.7)	14609 (16.9)	<0.01
Hypertension	727 (50.0)	2049 (43.6)	36653 (42.5)	<0.01
Beta blockers	382 (26.3)	1144(24.3)	20868 (24.2)	<0.01
Statins	263 (18.1)	451 (9.6)	968 (11.2)	<0.01
ACEs	369 (25.4)	1089 (23.2)	17252 (20.0)	<0.01
ARBs	161 (11.1)	539 (11.5)	8799 (10.2)	<0.01
Diuretics	200 (13.7)	689 (14.7)	12718 (14.7)	<0.01
Oral corticosteroids	398 (27.4)	1202 (25.6)	24177(28)	<0.01
Number of GP contacts	11.9 (12.6)	10.2 (10.7)	10.3(9.2)	<0.01

Data are numbers (%) of subjects unless otherwise stated.

BMI = Body Mass Index; BPH = Benign Prostatic Hyperplasia; COPD = Chronic Obstructive Pulmonary Disease; ACEs = Angiotensin converting enzyme inhibitors; ARBs = Angiotensin receptor blocker; GP = General Practitioner; OPD = Out-patient Department. Data show mean (standard deviation; SD) unless otherwise stated. IQR = Interquartile range. * Excluding missing data. NA= Not Available

Supplementary Table S4: Survival analysis of CPRD Cohort, with a time-dependent variable of BMI

	Primary analysis	
	Adjusted HR	*95% CI*
Finasteride alone vs Tamsulosin alone	*1.26*	*1.10 to 1.45*
Dutasteride alone vs Tamsulosin alone	*1.31*	*1.07 to 1.60*
Dutasteride alone vs Finasteride alone	*1.06*	*0.86 to 1.30*

BMI = Body Mass Index; HR= Hazard Ratio; CI = Confidence Interval

Assessment of confounding by tamsulosin

To address the validity of tamsulosin as the control and assess whether it independently influenced metabolic health, the incidence of T2DM in men receiving tamsulosin versus BPH patients undergoing transurethral resection of the prostate (TURPS) (Read Codes, Table S1a) were compared and analysis performed as in the main text, with propensity score matching (±0.05) in a ratio of 1:1.

Results: CPRD

Patients receiving TURP (Table S5) had suffered BPH for longer. This difference remained following propensity matching (1:1, n=1211 each group, Table S5), which did not eliminate all differences between groups e.g. the tamsulosin group received more statins. The risk of T2DM was not different (tamsulosin, adjusted HR: 0.71 (95%CI 0.46 to 1.11) vs TURPS) between groups following propensity scoring.

Results: NHIRD

Patient characteristics in the TURP group are in Tables S6. Again, these groups were harder to match by propensity scoring. The patients in the tamsulosin group were at less risk (0.86 (95%CI 0.77 to 0.96)) when compared with patients in the TURP group following propensity scoring.

Table S5. Baseline characteristics of Tamsulosin and TURP cohorts (UK CPRD database), before and after propensity matching

	Before Propensity score matching				After Propensity score matching		
	Tamsulosin N=16270	TURP N=1223	P value		Tamsulosin N=1211	TURP N=1211	P value
Age (y) mean (SD (y))	69.1 (10.3)	70.2 (9.3)	<0.01		66.7 (11.0)	70.2 (9.3)	<0.01
BMI category* (kg/m^2)			<0.01				0.02
1 (<18.5)	91 (0.6)	7 (0.8)			6 (0.6)	7 (0.8)	
2 (18.5-25)	4794 (33.6)	347 (41.2)			353 (34.4)	343 (41.0)	
3 (25-30)	6846 (48.1)	368 (43.5)			509 (49.7)	363 (43.4)	
4 (>30)	2518 (17.7)	124 (14.7)			157 (15.3)	123 (14.7)	
Smoking status*			<0.01				0.18
1 – Yes	1859 (11.8)	114 (11.9)			157 (13.5)	112 (11.8)	
2 – No	7485 (47.4)	467 (48.8)			589 (50.7)	461 (50.7)	
3 – Ex	6458 (40.9)	377 (39.3)			416 (35.8)	373 (39.4)	
Physical activity*			<0.01				0.57
1 – Inactive	699 (9.6)	37 (7.9)			59 (10.4)	37 (8.0)	
2 – Gentle	2636 (34.1)	156 (33.4)			290 (50.9)	236 (51.1)	
3 – Moderate	3917 (50.7)	241 (51.6)			35 (6.1)	33 (7.1)	
4 – Vigorous	473 (6.1)	33 (7.1)			186 (32.6)	156 (33.8)	
Alcohol consumption*			<0.01				0.54
1 – Yes	12233 (84.4)	745 (84.1)			896 (85.5)	734 (84.0)	
2 – No	1850 (12.8)	116 (13.1)			129 (12.3)	115 (13.2)	
3 – Ex	416 (2.9)	25 (2.8)			23 (2.2)	25 (2.9)	
Duration of BPH (days) median (IQR)*	56 (27-892)	478 (108-1884)	<0.01		63 (28-1038)	483 (108-1891)	<0.01
Disease history							
COPD	1169 (7.2)	62 (5.1)	<0.01		72 (6.0)	62 (5.1)	0.37
Dyslipidaemia	2711 (16.7)	144 (11.8)	0.55		213 (17.6)	142 (11.7)	<0.01
Hypertension	5241 (32.2)	404 (33.0)	<0.01		366 (30.2)	402 (33.2)	0.12
Beta blockers	3471 (21.3)	201 (16.4)	<0.01		214 (17.7)	198 (16.4)	0.39
Statins	5871 (36.1)	259 (21.2)	<0.01		380 (31.4)	257 (21.2)	<0.01
ACES	3844 (23.6)	231 (18.9)	<0.01		224 (18.5)	228 (18.8)	0.83
ARBS	1359 (8.4)	52 (4.3)	<0.01		92 (7.6)	52 (4.3)	<0.01
Diuretics	3669 (22.6)	244 (20.0)	0.04		209 (17.3)	242 (20.0)	0.09
Oral corticosteroids	1497 (9.2)	75 (6.1)	<0.01		95 (7.8)	74 (6.1)	0.09
Number of GP contacts mean (SD) (3 m before or after index date)	10.0 (2.4)	8.0 (4.6)	<0.01		9.1 (2.9)	7.9 (4.6)	<0.01

Data are numbers (%) of subjects unless otherwise stated. BMI = Body Mass Index; BPH = Benign Prostatic Hyperplasia; COPD = Chronic Obstructive Pulmonary Disease; ACEs = Angiotensin converting enzyme inhibitors; ARBs = Angiotensin receptor blocker; GP = General Practitioner; OPD = Out-patient Department. Data show mean (standard deviation; SD) unless otherwise stated. IQR = Interquartile range. * Excluding missing data.

Table S6. Baseline characteristics of Tamsulosin and TURP cohorts (NHIRD database), before and after propensity matching

	Before Propensity score matching			After Propensity score matching		
	**Tamsulosin N=86263**	**TURP N=2260**	**P value**	**Tamsulosin N=2260**	**TURP N=2260**	**P value**
Age (y) mean (SD (y))	65.7 (11.6)	71.1 (8.5)	<0.01	73.3 (10.1)	71.1 (8.5)	<0.01
BMI category* (kg/m^2)						
1 (<18.5)	NA	NA	NA	NA	NA	NA
2 (18.5-25)	NA	NA	NA	NA	NA	NA
3 (25-30)	NA	NA	NA	NA	NA	NA
4 (>30)	NA	NA	NA	NA	NA	NA
Smoking status*						
1 – Yes	NA	NA	NA	NA	NA	NA
2 - No	NA	NA	NA	NA	NA	NA
3 - Ex	NA	NA	NA	NA	NA	NA
Physical activity*						
1 - Inactive	NA	NA	NA	NA	NA	NA
2 – Gentle	NA	NA	NA	NA	NA	NA
3 – Moderate	NA	NA	NA	NA	NA	NA
4 – Vigorous	NA	NA	NA	NA	NA	NA
Alcohol consumption*						
1 – yes	NA	NA	NA	NA	NA	NA
2 – No	NA	NA	NA	NA	NA	NA
3 – Ex	NA	NA	NA	NA	NA	NA
Duration of BPH (days) median (IQR)*	21 (39-724)	207(66-1370)	<0.01	39 (22-405)	207 (66-1370)	<0.01
Disease history						
COPD	7491(8.7)	215 (9.5)	<0.01	278 (12.3)	215 (9.5)	<0.01
Dyslipidaemia	14609 (16.9)	303 (13.4)	<0.01	353 (15.6)	303 (13.4)	0.02
Hypertension	36653 (42.5)	1015 (44.9)	<0.01	1171 (51.8)	1015 (44.9)	<0.01
Beta blockers	20868 (24.2)	486 (21.5)	<0.01	615 (27.2)	486 (21.5)	<0.01
Statins	968 (11.2)	208 (9.2)	<0.01	215 (9.5)	208 (9.2)	0.18
ACES	17252 (20.0)	373 (16.5)	<0.01	461 (20.4)	373 (16.5)	<0.01
ARBS	8799 (10.2)	185 (8.2)	<0.01	194 (8.6)	185 (8.2)	0.22
Diu	12718 (14.7)	357 (15.8)	<0.01	330 (14.6)	357 (15.8)	0.05
Oral corticosteroids	24177 (28)	581 (25.7)	<0.01	660 (29.2)	581 (25.7)	<0.01
Number of OPD visits mean (SD) (3 m before or after index date)	10.3(9.2)	12.0 (10.4)	<0.01	10.3 (10.2)	12.0 (10.4)	<0.01

Data are numbers (%) of subjects unless otherwise stated. BMI = Body Mass Index; BPH = Benign Prostatic Hyperplasia; COPD = Chronic Obstructive Pulmonary Disease; ACEs = Angiotensin converting enzyme inhibitors; ARBs = Angiotensin receptor blocker; GP = General Practitioner; OPD = Out-patient Department. Data show mean (standard deviation; SD) unless otherwise stated. IQR = Interquartile range. * Excluding missing data. NA = Not Available.
